# 非小细胞肺癌患者肿瘤组织和外周血血浆中ENO1蛋白水平分析

**DOI:** 10.3779/j.issn.1009-3419.2010.12.02

**Published:** 2010-12-20

**Authors:** 莹 张, 敏 李, 宇 刘, 迺珺 韩, 开泰 张, 汀 肖, 书钧 程, 燕宁 高

**Affiliations:** 100021 北京，中国医学科学院北京协和医学院肿瘤医院肿瘤研究所“癌发生及预防分子机理”北京市重点实验室 Beijing Key Laboratory for Carcinogenesis and Cancer Prevention, Cancer Institute (Hospital), Peking Union Medical College & Chinese Academy of Medical Sciences, Beijing 100021, China

**Keywords:** 肺肿瘤, 烯醇化酶1, 血浆, 酶联免疫吸附分析, Lung neoplasms, ENO1, Plasma, ELISA

## Abstract

**背景与目的:**

肺癌严重威胁人类生存健康，有效的肿瘤标志物可以辅助诊断、判断预后和指导治疗。本研究旨在检测非小细胞肺癌患者肿瘤组织和血浆中烯醇化酶1（alpha-enolase, ENO1）蛋白水平，初步探讨ENO1作为肺癌相关蛋白标志物的可能性。

**方法:**

采用Western blot方法检测16例肺鳞癌患者的肿瘤组织及其配对正常肺组织中ENO1蛋白水平。采用双抗体夹心酶联免疫吸附分析（enzyme-linked immunosorbent assay, ELISA）测定42例健康体检者、34例肺部良性疾病患者和84例非小细胞肺癌患者三组人群血浆中ENO1蛋白水平。

**结果:**

在87.5%（14/16）的肺鳞癌患者肿瘤组织中ENO1蛋白表达量高于其配对正常肺组织；非小细胞肺癌患者血浆中ENO1蛋白水平高于健康体检者（*P*=0.031）和肺部良性疾病者（*P*=0.019），且ENO1蛋白在肺腺癌患者血浆中的水平高于肺鳞癌患者（*P*=0.023）。

**结论:**

非小细胞肺癌肿瘤组织和血浆中ENO1蛋白水平升高，提示ENO1可作为潜在的非小细胞肺癌相关血浆蛋白标志物。

## Abstract (en)

**Background and objective:**

Proper tumor markers are useful to diagnosis, prognosis and treatment for lung cancer. The aim of this study is to examine the levels of alpha-enolase (ENO1) protein in the tumor tissues and peripheral plasma samples obtained from non-small cell lung cancer (NSCLC) patients, and evaluate its potential clinical significance.

**Methods:**

The ENO1 protein levels in the tumor tissues and corresponding normal tissues from 16 cases of lung squamous cell carcinoma were analyzed by Western blot. The ENO1 protein levels in the plasma samples from 42 healthy individuals, 34 patients with lung benign disease and 84 patients with NSCLC were measured by double antibody sandwich enzyme-linked immunosorbent assay.

**Results:**

For 87.5% (14/16) of the patients with lung squamous cell carcinoma, the ENO1 protein level in the tumor tissues was higher than that in the corresponding normal lung tissues. The ENO1 protein level in the plasma of NSCLC patients was significantly higher than that in the plasma of healthy individuals (*P*=0.031) and patients with lung benign disease (*P*=0.019). Furthermore, the ENO1 protein level was significantly higher in the plasma of patients with lung adenocarcinoma than that of patients with lung squamous cell carcinoma.

**Conclusion:**

The elevated levels of ENO1 protein in the tumor tissues and the plasma samples from NSCLC patients indicate ENO1 may be a candidate biomarker of lung cancer.

在世界范围内，肺癌已经成为严重威胁人类生存健康的恶性肿瘤之一，其发病率和死亡率位列肿瘤首位^[1]^。中国国家卫生部公布的资料^[2]^显示，肺癌已替代肝癌成为我国首位恶性肿瘤死亡原因，占全部恶性肿瘤死亡的22.7%。肿瘤相关蛋白标志物是重要的辅助诊断、判断预后和指导治疗的生物学指标。目前，临床常规使用的肺癌相关血清学蛋白标志物主要包括癌胚抗原（carcinoembryonic antigen, CEA）、糖蛋白抗原125（carbohydrate antigen 125, CA125）、鳞状细胞癌相关抗原（squamous cell carcinoma antigen, SCC Ag）、细胞角蛋白19血清片段21-1（cytokeratin fragment antigen 21-1, CYFRA21-1）和神经元特异性烯醇化酶（neuron specific enolase, NSE），但其敏感性和特异性不高。

烯醇化酶1（alpha-enolase, ENO1）与NSE同属于烯醇化酶家族，位于细胞质，是糖酵解途径重要的代谢酶。近年来的研究^[3]^表明ENO1的细胞定位和生物学功能具有多样性，在肿瘤发生发展过程中发挥着重要作用，如定位于细胞膜的ENO1可促进肿瘤细胞的侵袭和转移^[4]^，定位于细胞质的ENO1可促进肿瘤细胞的生长和运动^[4]^，定位于细胞核的ENO1可抑制肿瘤细胞的生长^[5]^。Li和Chang等^[6, 7]^发现ENO1作为肺癌相关抗原，在肺癌细胞中高表达，并且其高表达提示临床预后差^[7]^。目前，尚未见报道ENO1在肿瘤患者外周血中的检测情况。

我们实验室利用新型的蛋白质组研究体系建立了一个肺癌相关分泌/释放蛋白数据库^[8]^，其中包括ENO1；从而我们推测ENO1有可能出现在肺癌患者外周血中。以上述发现为基础，在本项研究中我们分别采用Western blot方法和双抗体夹心酶联免疫吸附分析（enzyme-linked immunosorbent assay, ELISA）检测非小细胞肺癌患者肿瘤组织和血浆中ENO1蛋白的水平，并初步探讨ENO1作为肺癌相关蛋白标志物的可能性。

## 1 材料与方法

### 1.1 材料

所有临床样品取自1999年12月-2005年9月中国医学科学院肿瘤医院胸部肿瘤外科收治的患者。根据世界卫生组织（World Health Organization, WHO）2004年肺癌组织学分型标准进行肺癌组织学分型，根据国际抗癌联盟（International Union Against Cancer, UICC）2002年发布的第6版肺癌TNM分期系统进行肺癌分期。

#### 1.1.1 组织样品

组织样品取自接受手术治疗的16例男性肺鳞癌（squamous cell carcinoma, SCC）患者，中位年龄65岁（44岁-76岁）；其中Ⅰ期4例，Ⅱ期3例，Ⅲ期7例，Ⅳ期1例，无明确分期1例。所有患者术前均未接受过放疗或化疗。手术后收集切除的肿瘤组织及其配对的远端正常肺组织，-80 ℃保存。

#### 1.1.2 血浆样品

用于ELISA的血浆样品共160例，分别取自42例健康体检者，中位年龄52岁（40岁-65岁），男性30例，女性12例；34例肺部良性疾病患者，中位年龄51岁（14岁-70岁），男性23例，女性11例；84例非小细胞肺癌患者，中位年龄60岁（37岁-76岁），男性60例，女性24例；包括32例肺鳞癌和52例肺腺癌（adenocarcinoma, ADC），其中Ⅰ期31例，Ⅱ期26例，Ⅲ期22例，Ⅳ期5例。所有患者在采血前均未接受过放疗或化疗。

### 1.2 方法

#### 1.2.1 组织蛋白抽提和保存

取适当大小的组织块，加入300 L细胞裂解液（包含RIPA裂解缓冲液、蛋白酶抑制剂cocktail和PSMF），置于冰上裂解30 min，其间在涡旋器上进行充分混合；然后以12 000 rpm于4 ℃离心20 min，收集上清液，采用BCA Protein Assay Kit（Pierce, IL, USA）进行蛋白定量；分装后于-80 ℃保存。

#### 1.2.2 血浆的收集和保存

用EDTA抗凝管采集4 mL静脉血，于4 ℃离心，1 000 g、10 min，收集血浆，分装后于-80 ℃保存。

### 1.3 Western blot检测组织样品中ENO1蛋白水平

采用10% SDS-PAGE分离胶电泳分离变性后的60 g组织蛋白，随后使用半干式电转仪将分离后的蛋白转移至硝酸纤维素膜，电转后的膜在5%脱脂奶粉中室温封闭1 h。将转有组织蛋白的膜在鼠抗人ENO1单克隆抗体（1:1 000稀释，Edward F. Plow教授惠赠）溶液中4 ℃孵育过夜，然后在带辣根过氧化物酶标签的羊抗鼠二抗溶液（1:4 000稀释，Jackson ImmunoResearch，PA，USA）中室温孵育1 h。最后采用Western Blotting Luminol Reagent（Santa Cruz Biotechnology, CA, USA）发光试剂盒检测，X线胶片曝光、显影、定影。以β-actin作为内参蛋白，使用β-actin鼠单抗（1:3 000, Sigma-Aldrich, MO, USA）作为一抗。

### 1.4 双抗体夹心ELISA测定血浆中ENO1蛋白水平

以上述ENO1鼠单抗（1:100稀释，Edward F. Plow教授惠赠）为捕获抗体，包被96孔板，4 ℃过夜后，以2%BSA封闭96孔板4 h，之后每孔加入50 L待测血浆孵育1 h。以ENO1兔抗人ENO1多克隆抗体（1:4 000稀释，AVIVA Systems Biology，San Diego，CA）为检测抗体，孵育1 h。然后加入带辣根过氧化物酶标签的羊抗兔二抗溶液（1:4 000稀释，Jackson ImmunoResearch，PA，USA），孵育30 min。加入显色液，避光反应30 min，最后由酶标仪（Bio-Rad Laboratory, CA, USA）于450 nm/570 nm双波长设定下读取光密度（optical density, OD）值。由于目前没有商业化的ENO1标准蛋白，故采用肺癌细胞系A549的总蛋白用1× PBS梯度稀释，稀释比依次为1:20、1:40、1:80、1:160、1:320、1:640，以此绘制标准曲线，进行板间差校正。

### 1.5 实验数据的统计学处理

采用SPSS 11.5软件（SPSS INC, IL, USA）进行统计学分析。利用非参数检验中的*Mann-Whitney Test*比较两组人群血浆ENO1蛋白水平。*P* < 0.05为差异具有统计学意义。

## 2 结果

### 2.1 ENO1蛋白在肺鳞癌组织中的表达

ENO1蛋白在16例肺鳞癌患者的肿瘤组织及其配对正常肺组织中的表达情况如图 1所示，14例肺鳞癌患者肿瘤组织中ENO1的蛋白表达量高于其配对正常肺组织（除了17号患者和18号患者），高表达率为87.5%。

**1:**
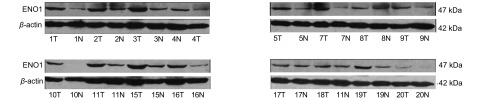
ENO1在肺鳞癌患者的肿瘤组织（T）及其配对正常肺组织（N）中的表达情况。*β*-actin作为内参蛋白。 ENO1 protein expression in tumor tissues and corresponding normal tissues from 16 cases of lung squamous cell carcinoma. *β*-actin served as an internal control of protein loading.

### 2.2 ENO1蛋白在不同人群血浆中的水平

采用双抗体夹心ELISA方法检测ENO1蛋白在三组人群（即健康体检者、肺部良性疾病患者和非小细胞肺癌患者）血浆中的水平。结果如表 1和图 2，非小细胞肺癌患者血浆中ENO1蛋白水平显著高于健康体检者（*P*=0.031）和肺部良性疾患者（*P*=0.019）。血浆中ENO1蛋白水平在肺部良性疾病患者和健康体检者之间没有明显差异（*P*=0.904）。肺腺癌患者血浆中ENO1蛋白水平明显高于肺鳞癌患者（*P*=0.023）。

**1:** 健康体检者、肺部良性疾患者、非小细胞肺癌患者三组人群血浆中ENO1蛋白水平 Levels of ENO1 protein in the plasma of non-small cell lung cancer patients and the controls

Diagnostic category	*n*	ENO1 protein level	*P*
		Median (interquartile range) (OD_450_/OD_570_)	
Healthy group	42	0.024 (0.038)	
Benign disease	34	0.022 (0.033)	0.904^a^
NSCLC	84	0.037 (0.053)	0.031^a^
			0.019^b^
SCC	32	0.036 (0.047)	0.023^c^
ADC	52	0.039 (0.055)	
a: compared with healthy group; b: NSCLC versus benign disease; c: SCC versus ADC; NSCLC: non-small cell lung cancer; SCC: squamous cell carcinoma; ADC: adenocarcinoma.			

**2:**
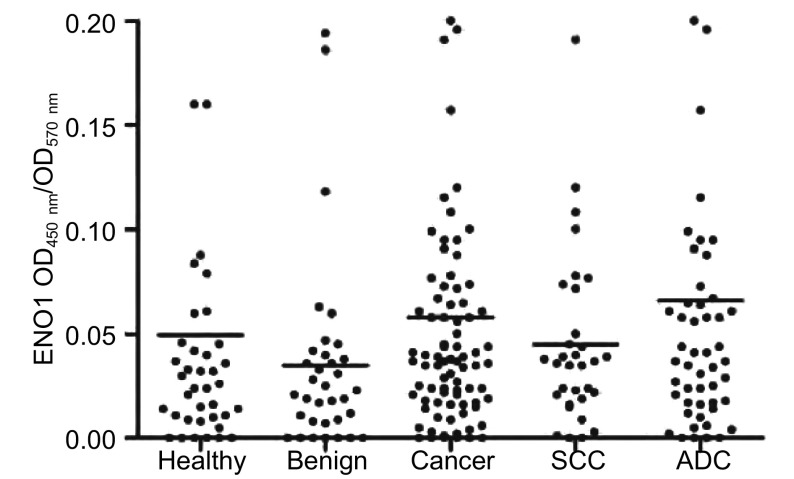
肺癌及其对照组人群血浆中ENO1蛋白水平。水平线代表ENO1蛋白水平的中位值。 Scatter plots of ENO1 protein levels in NSCLC cohort (cancer), benign disease cohort (benign) healthy women cohort (healthy), lung squamous cell carcinoma (SCC) cohort and lung adenocarcinoma (ADC) cohort. Median levels of ENO1 protein levels are represented by horizontal lines.

## 3 讨论

糖酵解途径是机体获取能量的一种方式，在供氧不足的生理状态下，它为机体提供能量。肿瘤细胞普遍存在糖酵解途径的增强^[9-11]^，Altenberg等^[12]^综合分析了肿瘤的糖酵解通路中代谢酶相关基因的表达情况，发现在24种肿瘤中糖酵解通路增强，肺癌是其中之一。糖酵解通路中涉及10个代谢酶，其中丙酮酸激酶（pyruvate kinase, PK）是糖酵解通路中的限速酶，其亚型PKM2除了发挥催化能量代谢的作用，还具有与肿瘤发生发展相关的生物学功能^[13, 14]^，并且肿瘤患者血浆中PKM2的水平显著升高^[15-17]^，提示其可能作为血浆诊断标志物。因此，糖酵解通路中其它代谢酶可能也与肿瘤有着紧密的联系。

烯醇化酶是糖酵解途径中的代谢酶，该家族包含三个成员，分别是ENO1、ENO2和NSE。NSE作为肺癌相关血清学蛋白标志物，已被用于临床上指导小细胞肺癌患者的疗效监测、复发预测和预后评估。我们实验室前期建立的肺癌相关分泌/释放蛋白数据库中包含与NSE同家族的ENO1^[8]^，作为肺癌相关抗原^[6, 7]^，ENO1极可能是潜在的肺癌标志物。本研究结果显示，ENO1蛋白在肺鳞癌患者肿瘤组织中的表达高于其配对正常肺组织，此结果与其它针对临床肿瘤样本的研究结果基本一致^[6, 7, 18]^，尽管与Chang等^[19]^的发现相反。

ENO1位于细胞质，它可能通过肿瘤细胞坏死和细胞更新（cell turnover）或者非经典的分泌途径被释放到细胞外^[20, 21]^，从而进入外周血。如糖酵解途径中大多数的代谢酶，包括ENO1，均可借助外来体途径（exosome pathway）被分泌到细胞外^[21]^。结果显示，较之健康人群和肺部良性疾病人群，非小细胞肺癌患者血浆中ENO1蛋白水平明显升高，因此ENO1可能作为肺癌相关血浆蛋白标志物。同时需要指出的是，因为目前尚无商业化的ENO1标准蛋白，所以我们无法绘制标准曲线来获得具体的蛋白浓度值。尽管ENO1可通过特定途径进入外周血，但其在外周血中的浓度很低，采用自行构建的双夹心ELISA体系检测的信号值偏低（OD值< 0.1）。不过，鉴于A549细胞内的ENO1蛋白的OD值最大值仅在0.3左右，所得血浆ENO1蛋白OD值亦为合理范围。由于目前尚未见采用ELISA方法检测外周血血浆中ENO1蛋白的报道，我们自行建立的ENO1蛋白ELISA检测体系及其实验结果具有一定的意义和参考价值。

ENO1对肿瘤进展具有双向作用，如定位于细胞膜的ENO1可促进肿瘤细胞的侵袭和转移^[4]^，定位于细胞质的ENO1可促进肿瘤细胞的生长和运动^[4]^，定位于细胞核的ENO1可抑制肿瘤细胞的生长^[5]^。但我们结果提示ENO1可能在肺癌的发生发展过程中更多地发挥着促进的作用。另外，我们还发现非小细胞肺癌患者血浆中ENO1蛋白水平与肺癌的组织病理类型相关，即肺腺癌患者血浆中ENO1蛋白水平显著高于肺鳞癌患者，推测ENO1作为潜在的肺腺癌血浆蛋白标志物可能更具有临床应用价值；因而有必要继续深入研究。
